# Calcitonin Gene-Related Peptide in Tension-Type Headache

**DOI:** 10.1100/tsw.2002.808

**Published:** 2002-06-07

**Authors:** M. Ashina

**Affiliations:** Department of Neurology and Danish Headache Center, Glostrup Hospital, University of Copenhagen, DK-2600 Glostrup, Copenhagen, Denmark

**Keywords:** tension-type headache, calcitonin gene-related peptide

## Abstract

In the last 10 years there has been increasing interest in the role of calcitonin gene-related peptide (CGRP) in primary headaches. Tension-type headache is one of the most common and important types of primary headaches, and ongoing nociception from myofascial tissues may play an important role in the pathophysiology of this disorder. CGRP sensory fibers are preferentially located in the walls of arteries, and nerve fibers containing CGRP accompany small blood vessels in human cranial muscles. It is well established that nociception may lead to release of CGRP from sensory nerve endings and from central terminals of sensory afferents into the spinal cord. It has also been shown that density of CGRP fibers around arteries is increased in persistently inflamed muscle. These findings indicate that ongoing activity in sensory neurons in the cranial muscles may be reflected in changes of plasma levels of neuropeptides in patients with chronic tension-type headache. To explore the possible role of CGRP in tension-type headache, plasma levels of CGRP were measured in patients with chronic tension-type headache. This study showed that plasma levels of CGRP are normal in patients and unrelated to headache state. However, the findings of normal plasma levels of CGRP do not exclude the possibility that abnormalities of this neuropeptide at the neuronal or peripheral (pericranial muscles) levels play a role in the pathophysiology of tension-type headache. Investigation of CGRP in other compartments with new sensitive methods of analysis is necessary to clarify its role in tension-type headache.

## INTRODUCTION

Chronic tension-type headache is one of the most common and important types of primary headaches[1,2] and represents a considerable health and socioeconomic problem[3]. Increased tenderness of pericranial myofascial tissues to manual palpation is the most prominent abnormal finding in patients with chronic tension-type headache[4,5,6,7,8]. Painful impulses from these tissues may be referred to the head and perceived as headache, and myofascial mechanisms may, therefore, play a major role in the pathophysiology of tension-type headache[9]. Substantial experimental evidence indicates that central sensitization, i.e., increased excitability of neurons in the CNS, generated by prolonged nociceptive input from the pericranial myofascial tissues, plays an important role in the pathophysiology of chronic pain[10] and chronic tension-type headache[11]. Various neuropeptides are involved in generation of painful input from the myofascial tissues and in the process of central sensitization[12].

In the last 10 years there has been increasing interest on the role of neuropeptides in primary headaches. Particularly, a role for calcitonin gene-related peptide (CGRP) has been implicated in the pathophysiology of neurovascular primary headaches such as migraine[13,14] and cluster headache[15,16]. However, the role of CGRP in generating pain in primary headaches is still unclear[14]. The present article presents a mini-review of the role of CGRP in tension-type headache based on our report for the 4th Annual Meeting on Calcitonin Gene-Related Peptide proceedings (*TheScientificWorld* (2001) 1(S1), 30).

## CGRP AND NOCICEPTION

CGRP, a 37–amino acid peptide, is one of the most abundant neuropeptides in the peripheral and central nervous system[17]. In rat muscle, sensory fibers containing CGRP are preferentially located in the wall of arteries[18], and sensory afferents expressing CGRP accompany small blood vessels in human cranial muscles[19]. The origin of circulating CGRP and the extent to which CGRP reaches the circulation or cerebrospinal fluid (CSF) from the peripheral or central nervous system are insufficiently known. In rats, the major part of circulating CGRP is released from perivascular nerve terminals[20,21]. It is well established that acute and chronic nociception leads to altered release of CGRP from sensory nerve endings and from central terminals of sensory afferents into the dorsal horn of the spinal cord[22,23,24,25]. It has also been shown that density of CGRP fibers around arteries is increased in persistently inflamed muscle[18]. Furthermore, it has been reported that CGRP is increased in the external jugular vein during stimulation of the trigeminal ganglion in humans by thermocoagulation[26]. Thus, possible changes of CGRP levels in blood may reflect changes in CGRP expression in the inflamed muscle tissue and the peripheral or central nervous system. Furthermore, these data suggest that CGRP is an important neurotransmitter or neuromodulator in the nervous system and that this neuropeptide may play a role in the process of central sensitization.

## CGRP IN CHRONIC TENSION-TYPE HEADACHE

The role of neuropeptides in chronic tension-type headache has previously been investigated in one study[27]. In this study Bach and colleagues reported normal CGRP levels in the CSF in patients with chronic tension-type headache[27]. However, it was not reported whether patients were examined during headache or in headache-free periods, or whether there was any relationship between CGRP levels and headache quality. To explore the possible role of CGRP in chronic tension-type headache, Ashina and colleagues measured plasma levels of CGRP[28] in the cranial and peripheral circulation of patients and controls. This study showed that plasma levels of CGRP are normal in both cranial and peripheral circulation of patients. Moreover, plasma levels of CGRP were largely unrelated to headache state[28]. Findings of normal plasma CGRP are particularly important because they are clearly different from our previous study in migraine patients with elevated levels of CGRP[14]. However, exploratory testing in relation to headache characteristics showed that eight patients with a pulsating pain quality, although fulfilling the International Headache Society (IHS)[29] criteria for tension-type headache and not for migraine, had higher plasma CGRP in the headache-free period than controls[28]. Plasma levels of CGRP in patients with predominantly pressing headache in the past did not differ from plasma GGRP in controls. In addition, there was no relationship between CGRP levels and muscular factors[28].

The question is how to explain increased plasma CGRP in chronic tension-type headache patients with pulsating headache quality. Since CGRP levels are increased in migraineurs[14] and in patients with pulsating headache[28] in the headache-free period, ongoing nociception from cephalic or extracephalic vasculature, or both, seems to be ruled out. Circulating CGRP may be involved in the regulation of blood flow and in the maintenance of vascular tone[30]. It is possible that increased interictal CGRP levels in patients with pulsating-headache–like migraines may reflect altered vascular control due to abnormal release of CGRP from sensory neurons[14]. Therefore, the most likely explanation is that these patients, although fulfilling the IHS criteria for chronic tension-type headache and not having any migraine history, are in fact pathophysiologically related to migraine. Although the finding of increased plasma CGRP in patients with pulsating headache quality is very interesting, one should be very cautious with interpretation of *post hoc* analysis, particularly with a small number of patients. Therefore, a prospective study with a large number of patients is required to confirm this observation.

## CONCLUDING REMARKS

Studies on the role of CGRP in chronic tension-type headache indicate that plasma levels of CGRP are normal in patients and largely unrelated to headache state[28]. Furthermore, findings of normal plasma CGRP add to the growing list of differences between migraine and tension-type headache. It is possible that changes of neuropeptide levels at the spinal/trigeminal level or in the myofascial tissue of these patients do not reach the cranial or peripheral circulation. It would have been optimal to measure CGRP levels in the pericranial muscles and CSF or even at the neuronal level. However, this is difficult for both practical and ethical reasons. Thus, the findings of normal levels of CGRP in the cranial and peripheral circulation do not exclude the possibility that abnormalities of these neuropeptides at the neuronal or peripheral (pericranial muscles) levels play a role in the pathophysiology of chronic tension-type headache. Investigation of neuropeptides in other compartments with new sensitive methods of analysis is necessary to clarify their role in chronic tension-type headache. Finally, future CGRP studies in chronic tension-type headache sufferers with pulsating pain quality and in migraine patients with pressing pain quality, but otherwise fulfilling the IHS migraine criteria, are required to clarify whether increased CGRP levels are associated with pulsating pain quality.

## ACKNOWLEDGMENTS

The study was supported by the Foundation of Jacob Madsen and his wife Olga Madsen, by the Foundation for Research in Neurology, and by the Danish Headache Society.
